# Genome-Wide Characterization and Linkage Mapping of Simple Sequence Repeats in Mei (*Prunus mume* Sieb. et Zucc.)

**DOI:** 10.1371/journal.pone.0059562

**Published:** 2013-03-28

**Authors:** Lidan Sun, Weiru Yang, Qixiang Zhang, Tangren Cheng, Huitang Pan, Zongda Xu, Jie Zhang, Chuguang Chen

**Affiliations:** 1 College of Landscape Architecture, National Engineering Research Center for Floriculture, Beijing Forestry University, Beijing, P. R. China; 2 Beijing Microread Genetics Co., Ltd, Beijing, P. R. China; Kansas State University, United States of America

## Abstract

Because of its popularity as an ornamental plant in East Asia, mei (*Prunus mume* Sieb. et Zucc.) has received increasing attention in genetic and genomic research with the recent shotgun sequencing of its genome. Here, we performed the genome-wide characterization of simple sequence repeats (SSRs) in the mei genome and detected a total of 188,149 SSRs occurring at a frequency of 794 SSR/Mb. Mononucleotide repeats were the most common type of SSR in genomic regions, followed by di- and tetranucleotide repeats. Most of the SSRs in coding sequences (CDS) were composed of tri- or hexanucleotide repeat motifs, but mononucleotide repeats were always the most common in intergenic regions. Genome-wide comparison of SSR patterns among the mei, strawberry (*Fragaria vesca*), and apple (*Malus*×*domestica*) genomes showed mei to have the highest density of SSRs, slightly higher than that of strawberry (608 SSR/Mb) and almost twice as high as that of apple (398 SSR/Mb). Mononucleotide repeats were the dominant SSR motifs in the three Rosaceae species. Using 144 SSR markers, we constructed a 670 cM-long linkage map of mei delimited into eight linkage groups (LGs), with an average marker distance of 5 cM. Seventy one scaffolds covering about 27.9% of the assembled mei genome were anchored to the genetic map, depending on which the macro-colinearity between the mei genome and Prunus T×E reference map was identified. The framework map of mei constructed provides a first step into subsequent high-resolution genetic mapping and marker-assisted selection for this ornamental species.

## Introduction

Belonging to the Rosaceae, sub-family Prunoideae, mei (*Prunus mume* Sieb. et Zucc., 2n = 2x = 16), originating in Southwestern China, is believed to have been cultivated in China for over 3000 years [1]. Because of its prominent ornamental characteristics, mei has now been widely cultivated in other East Asian countries including Korea, Japan and Vietnam [1], [2]. Mei possesses colorful corollas, varying types of flowers, and pleasant fragrance and is extensively grown as an early-blooming garden ornamental plant [2]. In particular, mei is characterized by its inherent tolerance to temperatures as low as −4°C [2]. This characteristic allows mei to flower in winter or early spring, while most other ornamental plants and fruit trees are still dormant [1], [2]. In addition, mei has an important value in Chinese traditional medicine by providing salted mei, mei liquor, mei juice, and mei sauce beneficial for human health [2]. Despite its importance, however, we have little knowledge about the genetic mechanisms that underlie biological and ornamental traits of mei. This situation has changed in recent years by use of DNA markers for mei genetic relatedness and diversity analyses [3]–[5]. However, efforts to implement selection and breeding for superior mei varieties have been impeded by the lack of sufficient user-friendly DNA markers utilized to construct a genetic map.

SSRs, or microsatellites, are conventionally defined as tandem repeats of short DNA sequences that are of two to six base pairs (bp) in length [6]. The current definition of SSRs includes mononucleotide repeats [7].There is no official strict minimum number of repeats that define a SSR, but it is accepted that 10 repeats are sufficient if the repeat motif is a mononucleotide, 6 are sufficient for dinucleotides, 4 for trinucleotides, and 3 repeats are sufficient for tetra-, penta-, and hexanucleotides [8], [9]. Because they are codominant, abundant, multi-allelic, highly reproducible, easily examined using automated procedures, and uniformly distributed over the whole-genome [10], SSRs are extensively used as the main markers for DNA fingerprinting, linkage mapping, marker-assisted selection, map-based cloning, and comparative genomics analyses across species [11]–[13]. With the advent of next-generation sequencing (NGS) platforms, faster and more cost-effective means of developing a huge number of SSR markers at the whole-genome scale has become feasible in many plants, such as rice [14], Brachypodium [9], and poplar [15]. Genomic SSRs, which are highly polymorphic and tend to be widely distributed throughout the genome, can be used to evaluate the distribution and frequency of different types of SSRs in the genome, facilitating analysis of SSR evolution and offering better map coverage than the conventional approaches used for the initial identification of SSRs, such as expressed sequence tags (ESTs) [16], and bacterial artificial chromosomes (BACs) [17]. Recently, following the sequencing of apple [18] and strawberry [19] genomes, mei has been sequenced in our lab using the Solexa platform [20]. The advances in the genetic study of the Rosaceae allow us to characterize the genome-wide frequency and distribution of SSRs and use them to construct a genetic linkage map for mei. Meanwhile, they can facilitate the study of evolutionary dynamics of SSR markers in Rosaceae.

In this article, we report the genome-wide characterization of SSRs in the mei genome and a comparative analysis of the pattern of SSRs among different species from the Rosaceae. A robust set of polymorphic SSRs was developed from the genomic sequences of mei to construct a framework genetic linkage map. The genetic map was used to construct a framework physical map by anchoring the scaffolds from the sequenced mei genome, and macrosyntenic relationships between the mei genome and the Prunus T×E reference map [an interspecific almond ‘Texas’×peach ‘Earlygold’ (here given as T×E) F_2_ mapping population] [21] were identified.

## Results and Discussion

### Characterization of SSRs in the Mei Genome

A total of 188,149 perfect SSRs, all with repeat motifs at least 10 bp long and all of which exactly matched specific single motifs in an uninterrupted fashion were detected [22], [23]. Their repeat units ranged in length from 1 bp to 8 bp, accounting for 1.2% of the total size (∼237 Mb) of the assembled mei genome [20] and occurring at an overall frequency of 794 SSR/Mb. The frequency of different types of SSRs was negatively correlated with the number of nucleotides; from the most frequent mononucleotide repeats (67,183, 35.7% of the total) exponentially decreasing to the least frequent octanucleotide repeats (899, only 0.5% of the total) (Table 1, Figure S1). Together, mono-, di-, tri-, and tetranucleotide repeats accounted for 87.9% of the total number of SSRs identified. In general, the frequency of SSRs decreased stepwise with increasing repeat unit length, with the exception of the frequency of trinucleotide repeats (12.9%), which was lower than that of tetranucleotide repeats (16.8%) (Table 1). The distribution and frequency of SSRs in mei genome was similar to the apple genome but different from the strawberry genome, which was consistent with the known phylogenetic distances between the three species [24]. Our research highlighted patterns of SSR composition which broke down with increasing evolutionary distance among organisms [25]. Differences in the effects of past selection pressures and mismatch repair mechanisms on specific motifs and regions in different plant genomes are also considered some of the main causes of these phenomena [9], [26]. We further examined the distribution of mei SSRs with respect to the number of repeat motifs. Although the frequency of SSRs decreased as the number of repeat motifs increased, the decline became notably sharper with tetra- to octanucleotide motifs, as indicated by the average number of these repeat motifs, which was only about one-fourth as many as mononucleotide motifs (Table 1, Figure S1). Varying frequency and distribution of SSRs can be explained from the major mechanism of SSR formation. Proto-microsatellites are created spontaneously from unique sequences by substitution or insertion [27], with subsequent elongation or expansion by transposable elements (TEs) [28]. In the mei genome, short motifs are more likely to form proto-microsatellites than long motifs, leading to the more frequent occurrence of low-copy than high-copy nucleotide repeats.

**Table 1:** Distribution of perfect SSRs in genomes of mei and other Rosaceae species.

Repeat type	Count			Relative frequency (%)			Mean repeat number			GC content (%)			Density (SSR/Mb)		
	Mei	Strawberry	Apple	Mei	Strawberry	Apple	Mei	Strawberry	Apple	Mei	Strawberry	Apple	Mei	Strawberry	Apple
Mononucleotide	67,183	47,094	96,740	35.7	36.2	39.9	13	13	12	3.8	4.6	3.8	284	220	159
Dinucleotide	42,291	30,050	52,929	22.5	23.1	21.8	11	11	10	34.2	31.2	29.6	178	140	87
Trinucleotide	24,237	21,864	30,921	12.9	16.8	12.7	5	5	5	32.6	40.2	35.4	102	102	51
Tetranucleotide	31,575	18,935	38,713	16.8	14.5	16.0	3	3	3	18.3	28.8	21.0	133	89	64
Pentanucleotide	12,289	5,956	13,602	6.5	4.6	5.6	3	3	3	22.5	31.6	27.1	52	28	22
Hexanucleotide	7,054	4,166	6,026	3.7	3.2	2.5	3	3	3	26.6	36.4	31.5	30	20	10
Heptanucleotide	2,621	1,826	2,778	1.4	1.4	1.1	3	3	3	21.2	30.3	25.7	11	9	5
Octanucleotide	899	307	977	0.5	0.2	0.4	3	3	3	25.9	22.7	30.9	4	1	2
Total	188,149	130,198	242,686	100	100	100	9	9	9	21.5	24.7	20.6	794	608	398

SSRs had an uneven distribution in the mei genome. While SSRs were abundant (896 SSR/Mb) in the intergenic regions, only 7,933 SSRs were found in its 36 Mb CDS region (220 SSR/Mb) (Table 2). Despite this low abundance, 73.2% (4,089) of the SSR-containing CDS could be assigned to one or more functional annotations [Gene ontology (GO) terms] [29], including all the three top-level ontologies, i.e., biological process, cellular component, and molecular function. Among these, 19,786 GO terms were categorized under biological process, 8,264 under cellular component, and 6,732 under molecular function (Table S1, Figure S2). Among biological process, the largest categories were cellular process (17.4%) followed by biological process (17.3%). The major portion of cellular component was from cellular component (18.4%) and cell (14.0%) categories. However, 2,818 (41.9%) of the molecular function genes were related to binding activity (Table S1, Figure S2). Information about the distribution and frequency of SSRs is valuable for discovering functional genes used for marker-assisted selection in mei breeding.

**Table 2:** Distribution of SSRs in CDS and intergenic regions of mei and other Rosaceae species.

	Mei			Strawberry			Apple		
Genome region	CDS	Intergenic	Total	CDS	Intergenic	Total	CDS	Intergenic	Total
Count	7,933	144,230	152,163	9,268	78,806	88,074	7,501	198,344	205,845
Relative frequency (%)	5.2	94.8	100	10.5	89.5	100	3.6	96.4	100
Size (Mb)	36	161	197	40	118	158	37	522	559
GC content (%)	50.0	20.3	21.5	52.9	23.1	25.9	53.3	19.5	20.6
Density (SSR/Mb)	220	896	772	232	668	557	203	380	368

### Genome-wide Comparison of SSRs among Mei and Related Rosaceae Species

The complete mei, strawberry, and apple genomes were used to compare SSR patterns among the Rosaceae (Table 1). Of these three genomes, mei showed the highest frequency of SSRs (794 SSR/Mb), nearly one-fourth higher than strawberry (608 SSR/Mb) and almost two times higher than apple (398 SSR/Mb). Because the apple genome is the largest (∼742 Mb) [18] and because it may have experienced double whole-genome duplication (WGD), the low density of SSRs in apples was considered consistent with the hypothesis that the frequency of SSRs in plants is negatively related to genome size [30]. This may indicate that the SSRs are underrepresented in the repetitive parts of the plant genome such as long terminal retrotransposons, which are considered to play important roles in genome expansion [30]. The higher SSR density relative to the larger genome size in mei (∼280 Mb) [20] than in strawberry (∼240 Mb) [19] may be indicative of a lower proportion of low-copy sequences in the latter [30]. This showed that there may be more low-copy sequences in the mei genome than in the apple and strawberry genomes.

In each of the three genomes, SSR repeats shorter than four nucleotides made up more than 85% of all the SSRs. The frequency of SSRs decreased with increasing length and number of repeat unit. Mononucleotide repeats were more common than other type of repeats. This was consistent with the observations from most other dicots analyzed including Arabidopsis, pigeon pea, cocoa, Chinese cabbage, and potato (Table 1, Table S2). Meglecz et al. suggested that SSR contents of organisms within clades that formed within the past 200 million years tended to be similar [25]. In this way, the relatively high correspondence of SSR coverage among Rosaceae and other eudicots could be explained by a recent radiation event or events [31].

A/T repeats were more common than C/G repeats in all three Rosaceae species and in other eudicots (Table S3). A/T rich repeats were ascribed to the poly-A tails of dispersed retroposed sequences such as long repetitive elements (LINEs) and processed pseudogenes [32]. AG/CT repeats were the most common dinucleotide motifs in all three Rosaceae species but not in the other eudicots (Table S3). We hypothesized that the accumulation of these repeats may have been promoted by special selection pressure after divergence of the Rosaceae from other eudicots. This was confirmed by a study showing that the patterns of the SSR composition from a common ancestor broke down after divergence [25]. As in other sequenced eudicots (Table S3), CG/CG repeats were scarce in the Rosaceae genomes. Among trinucleotide motifs, AAG/CTT and AAT/ATT repeats were the most common, whereas CCG/CGG repeats were the scarcest. AAAT/ATTT repeats were found to be the most common tetranucleotide motifs, and CCGG/CCGG and CCCG/CGGG repeats were rarer (Table S3). The low content of CG-rich motifs could be explained by the low GC content of SSRs, which was 9.8–24.7% (Table 1, Table S2) in Rosaceae and the other eudicots. Replication slippage, which causes microsatellite mutations, was responsible for this phenomenon [33]. According to this theory, motifs with hairpin structures and the self-complementary repeats like (CTG)_n_, (CCG)_n_, (AT)_n_, and (GC)_n_ are more readily accumulated in the genome [34], [35]. However, methylation of cytosine, which occurs predominantly at the CG dinucleotides, CHG (where H is A, C, or T), and CHH (where H is A, C, or T) sites in plants tends to decrease the frequency of GC-rich repeats [36]. Nonetheless, AT-rich repeat motifs were far rarer in CDS regions than in intergenic regions in the three Rosaceae species (Table S4, Table S5). For example, AT/AT and AAT/ATT repeat motifs in the CDS regions were rarer than in intergenic regions (3.7–10.3% vs. 38.8–45.8%; 0.5–1.7% vs. 15.2–35.1%) (Table S4, Table S5). One possible explanation for the biases of repeat motifs in these regions could be that the (A)_n_T repeat motifs are more sensitive to evolutionary constraints than other motifs and are removed from the CDS regions [37]. This was ascribed to the selection against the stop codon and to retention of the selection of mRNA stability by avoiding AU [37].

In addition to the AT-rich repeats, the overall density of SSRs in CDS was also much lower than in intergenic regions in Rosaceae genomes. The highest SSR density in CDS, which was observed in the strawberry genome, was only 232 SSR/Mb; the intergenic SSR densities were 380 SSR/Mb, 668 SSR/Mb, and 896 SSR/Mb in apple, strawberry and mei genomes, respectively (Table 2). The low SSR density in CDS regions of the genome can be attributed to the functional importance of these regions, which are believed to have experienced more negative selective pressure than intergenic regions [30]. Therefore, the SSR density of the CDS regions was far lower than that of intergenic regions. Possibly because of natural selection against frame-shift mutations, which limited the expansion of non-triplet SSRs [38], most SSRs in CDS were composed of tri- or hexanucleotide repeat motifs (Table S6). In intergenic regions, mononucleotide repeats were always the dominant SSR repeat units (Table S7).

In summary, as in many other studies [9], [25], our results indicated that the distribution and frequency of SSRs in Rosaceae was affected by several factors including special selection pressure, mutation mechanisms, and genome structure. The increasing availability of genome resources for Rosaceae species may allow the role of these SSRs in the divergence of Rosaceae to be more comprehensively elucidated.

### Construction of a SSR-based Genetic Linkage Map of Mei

Polyacrylamide gel (PAG) electrophoresis was used to survey 670 pairs of SSR primers among two mei varieties, *Prunus mume* ‘Fenban’ and *Prunus mume* ‘Kouzi Yudie’, and five randomly chosen segregating progeny from the cross between the two varieties (Table S8). The result showed that 648 primer pairs (96.7%), including 410 class I (hypervariable markers, consisting of SSRs ≥20 nucleotides in length) and 238 class II (potentially variable markers, consisting of SSRs ≥12 nucleotides and <20 nucleotides in length) primers, yielded unambiguous, stable PCR products with expected sizes (Figure S3, Table S8). Of these, 144 scorable polymorphic SSR primers, including 107 class I and 37 class II SSR primers that were labeled with fluorescent dyes, were used to detect segregated markers in the mapping population derived from ‘Fenban’ and ‘Kouzi Yudie’ (Figure S4, Table S8). The higher level of polymorphism for class I SSRs than class II SSRs was also detected in rice [14] and Brachypodium [9]. Dinucleotide repeats accounted for 57.0% of class I SSRs (Table S8), which was consistent with the high content of dinucleotide repeats (59.7%) in class I SSRs of the mei genome (Figure S1). This provides insight into the rapid and reliable extraction of additional polymorphic SSRs from dinucleotide repeats of class I SSRs in mei genome and facilitates the construction of a high-density genetic map.

**Figure 1:**
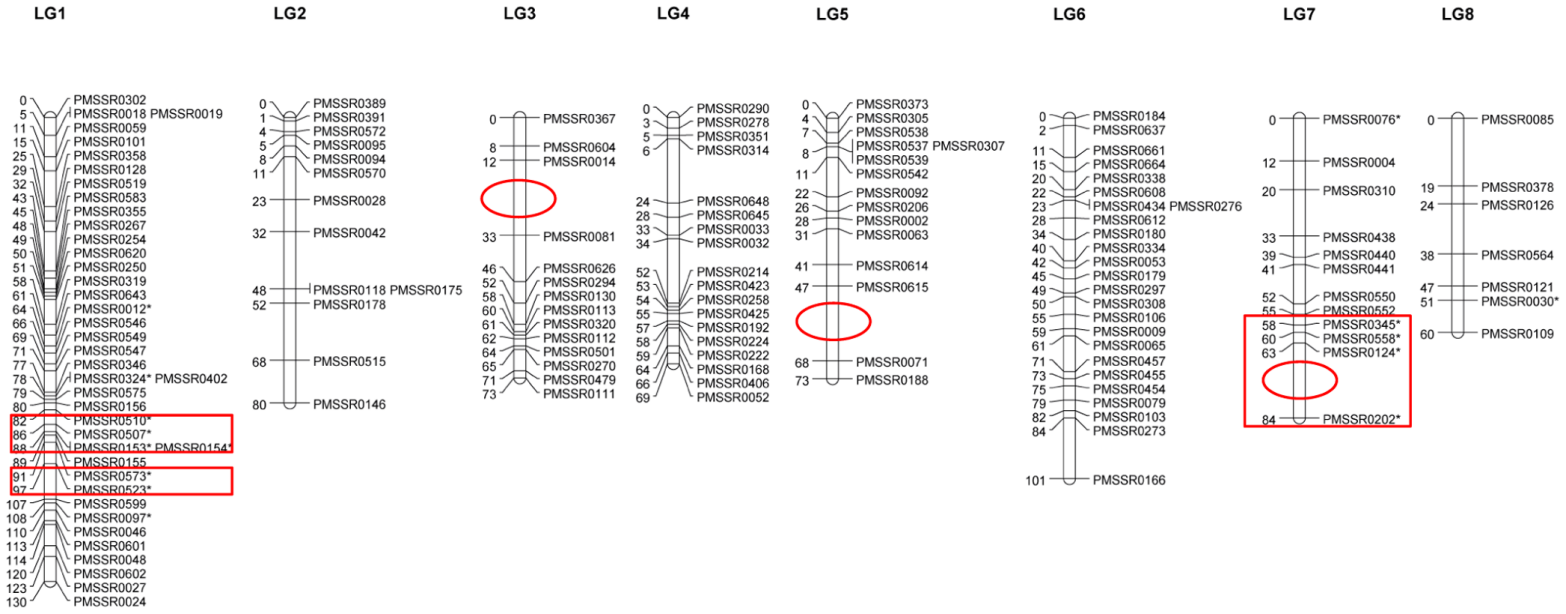
Framework genetic map of *P. mume* ‘Fenban’×*P. mume* ‘Kouzi Yudie’. Numbers to the left of each LG are marker positions identified in cM. Markers that show distorted segregation distributions are starred (*; *P*<0.05). Ovals indicated gaps are greater than 20 cM. SDRs are shown by boxes.

One hundred and twenty-nine (89.6%) of the 144 polymorphic markers exhibited standard Mendelian segregation (1∶1, 1∶2∶1 or 1∶1∶1∶1) and 15 (10.4%) showed significant segregation distortion (*P*<0.05). Using all markers including distorted markers, a framework linkage map was constructed, which included eight LGs, equal to the haploid chromosome number of mei (Figure 1). The number of markers ranged from 7 in LG8 to 40 in LG1, with an average of 18. The lengths of LGs ranged from 60 to 130 cM, totaling to 670 cM. The scaffolds that were anchored to each LG ranged in size from 5 to 15 Mb. The average marker interval was 5 cM (Table 3). The molecular marker distributions of individual LGs were uneven, which may be attributed to two causes. First, number of SSRs varies over mei chromosomes. It was observed that the number of SSRs in chromosome 2 (34,985) was about two times that in chromosome 6 (16,837) (Table S9) [20]. Chromosome 2 and chromosome 6 corresponded to LG1 and LG8, respectively. In published Prunus species genetic maps, the number of markers ranged from 3 to 21 in peach [39], and from 4 to 27 in apricot [40]. Second, this uneven distribution of SSR markers along each LG may be due to uneven marker polymorphisms and recombination rates. A similar result was also detected in Madagascar periwinkle [41] and groundnut [42].

**Table 3:** Marker distribution on the eight linkage groups of mei.

Linkage groups	No. of markerloci	Genetic distance (cM)	No. of anchored scaffolds	Scaffold size (Mb)	Average interval amongmarkers (cM/SSR)	Maximum gaps (cM)
LG1	40	130	13	15	3	11
LG2	13	80	8	8	6	16
LG3	14	73	7	10	5	21
LG4	18	69	9	5	4	18
LG5	15	73	9	7	5	21
LG6	25	101	14	11	4	17
LG7	12	84	6	5	7	21
LG8	7	60	5	5	9	19
Total	144	670	71	66	5	144

In the mei genome, four regions with a high marker density stemming from suppressed recombination were detected, distributed in an interstitial region of LG1, LG3 and LG4 (Figure 1). In other plant species, such as papaya [43] and asparagus bean [44], a similar pattern was also reported. The marker-rich regions are generally associated with pericentromeric or heterochromatin regions of the mei chromosome [43]. Near the end of LG3, LG5, and LG7, there was a marker interval of >20 cM (Figure 1, Table 3). This may be due to higher levels of recombination at terminal regions of mei chromosomes, as also detected in asparagus bean [44] and watermelon [45]. These regions were enriched in recombinationally active chromosome ends, which indicated that they may be the most telomere- and gene-rich regions in mei genome [46]. However, the gaps may also be because of a shortage of markers detected in these regions.

Of 15 distorted markers, ten showed clustered distribution, of which six were located on LG1 and four on LG7. The clustering of DNA markers showing distorted segregation has been widely reported in many plants. It usually takes place on the so-called segregation distortion regions (SDRs) of the LGs [44], [47]. The two SDRs in LG1 were adjacent to each other but were separated from the third one in LG7 (Figure 1). Marker distortion may be due to preferential selection [48] and/or contain sub-lethal genes causing gamete transmission deviation [49]. The SDR in LG7 may not affect the calculation of map distance ascribed to the presence of only one gametophytic factor on the chromosome [50]–[52]. The map distance in LG1 may have deviated slightly from the true values, owing to the putative presence of two linked gametophytic factors on the chromosome [52].

### Syntenic Relationship between the Genomes of Mei and Prunus

**Figure 2:**
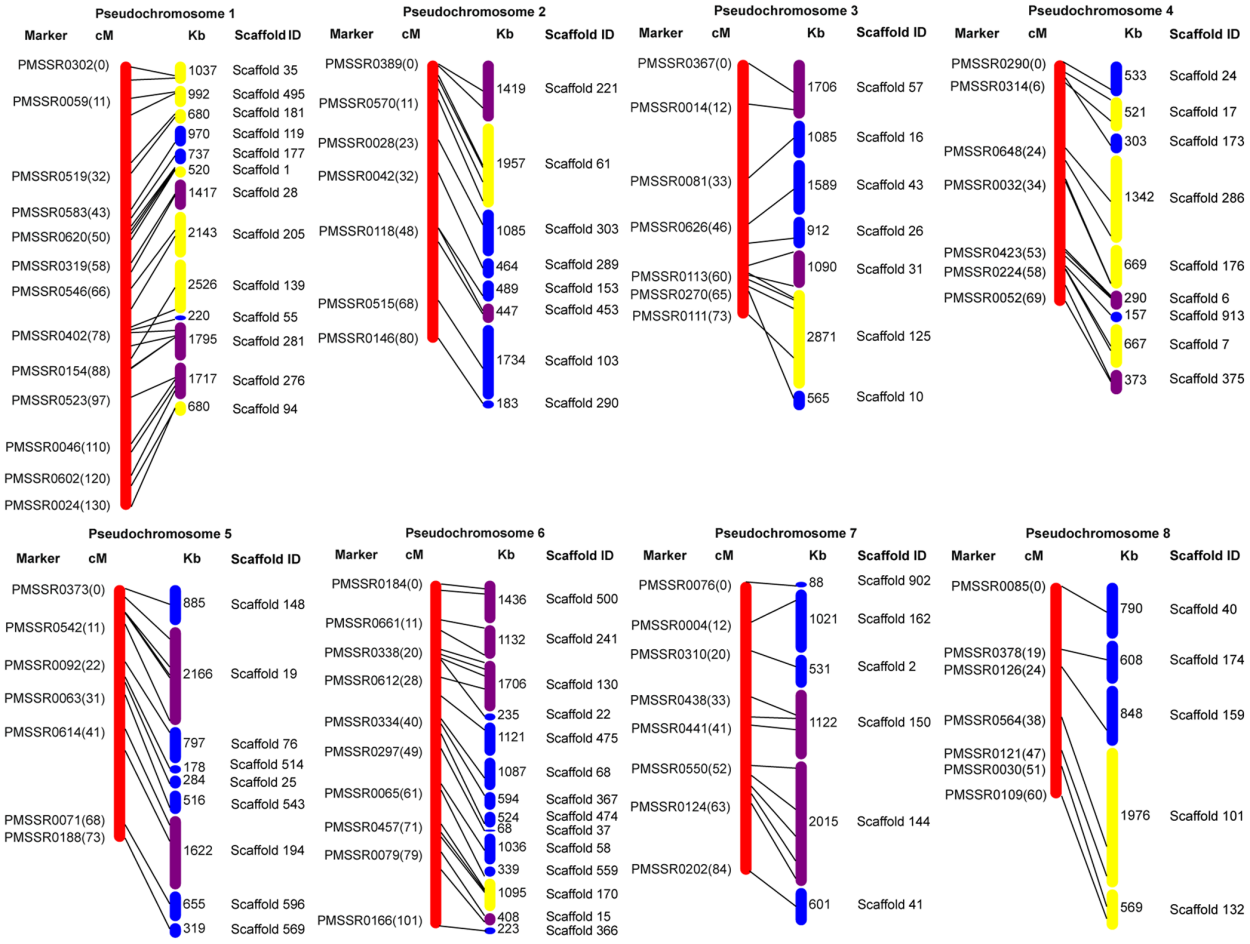
Anchoring the mei genome to the framework genetic map. Scaffolds representing 66 Mb of assembled sequences are mapped to the genetic map with 144 SSRs. Markers and genetic distances in cM are showed on the left. Scaffold names and lengths in Kb are showed on the right. Purple scaffolds are oriented in forward direction, yellow in reverse direction, and blue not oriented.

Comprehensive alignments of the interspecific Prunus T×E reference map and other linkage maps of peach, apricot, and cherry have revealed strong colinearity among these genomes [13], [53], [54]. However, the synteny of a mei linkage map with the Prunus reference map remains elusive. Here, we anchored 71 scaffolds (totaling to 66 Mb in length) of the mei genome to the Prunus T×E reference map, accounting for about 27.8% of the 237 Mb assembled mei genome [20] (Figure 2), with an average of 100 Kbp/cM (Table 3). In each scaffold, there were two markers on average. A total of 32 scaffolds (45.1%) had more than one marker, which were oriented on the genetic map (Table 3). From alignment analysis, the relative order of markers in linkage maps was detected to be basically consistent with their relative physical positions (Figure 2), suggesting that the framework genetic map can be used to estimate the relationship between physical distance and recombination fraction. In this sense, the linkage map can support physical map assembly and provides a useful resource for map-based gene isolation and comparative genome analysis.

However, a few sparse markers on LG1, LG3, LG4, and LG6 appeared in the genome sequence in an order that was locally inconsistent with the physical distance (Figure 2). Further investigation of the segregation pattern of scattered markers indicated discrepancies between their physically- and recombination-based positions. Such markers have also been observed in many organisms including papaya [55], strawberry [19] and watermelon [45]. These large genetic distances may represent relatively small physical distances in the high-recombination regions located in gene-dense regions and proximity to telomeres [45]. The small genetic distances may represent relatively large physical distances in recombination suppression regions located in pericentrometric or heterochromatic regions [45], [56]. The regions among markers located in the crossing lines of LG1, LG3, LG4, and LG6 showed small genetic distances representing large physical distances in recombination suppression regions. These regions are generally considered as pericentromeric or heterochromatic regions. These findings can be used to identify pericentromeric, telomere and gene-rich regions and further detect crossovers throughout the mei genome.

**Figure 3:**
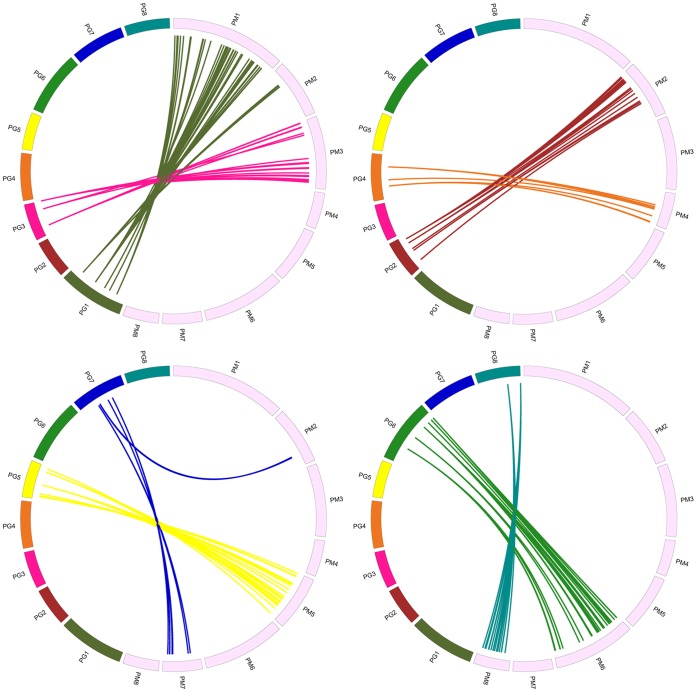
Comparative analysis between genomes of mei and Prunus. Comparison between mei genome (PM1 to PM8) and Prunus T×E reference map (PG1 to PG8) [21]. Different colors represent different pseudochromosomes between the genomes of mei and Prunus. Orthologous sequences are linked by solid lines. For visibility, the syntenic relationships are revealed by four circles, each showing the syntenies of two pairs of pseudochromosomes.

A high level of macro-colinearity was revealed by aligning the map positions of 192 polymorphic Rosaceae conserved ortholog set (RosCOS) markers anchored on a Prunus T×E reference bin map [21] and mei genome sequences anchored on the genetic map of 144 SSRs (Figure 3, Table S10). Complete syntenic relationships were observed between Prunus linkage groups (PG)2 and the mei pseudochromosome (PM)2. Another five pairs, PG3–PM3, PG4–PM4, PG5–PM5, PG6–PM6, and PG8–PM8 were detected. This high level of synteny strongly suggests marked genome conservation between mei and other Prunus species. These results were corroborated with other highly conserved syntenic relationships among the *Prunus* spp. and Prunus reference maps [13], [53], [54]. However, PG1 was syntenic to two mei pseudochromosomes, PM1 and PM2, and PG7 was syntenic to PM2 and PM7, which suggests that fission, fusion, and translocation events have occurred since the divergence of the two species [57]. Despite an overall high level of synteny between the mei genome and the Prunus T×E reference map, the colinearities were disrupted by chromosomal rearrangements involving two translocation events (Figure 3, Table S10). In contrast, only one translocation site related to G6 and G8 was detected between almond (cv. ‘Garfi’) and peach (cv. ‘Nemared’) by analyzing the F_2_ population [58]. This suggests that the mei genome may have experienced more complicated genome reshuffling events.

A closer look at the macro-colinearity based on marker positions demonstrated that PM1, PM3, PM4, PM5, PM6, PM7, and PM8 in mei may have not undergone chromosome rearrangement (Figure 3). Most of PM2 appeared to have originated from ancestral PG2. This chromosome may have received some fragment insertions from ancestral PG1 and PG7. The rearrangement breakpoints were found at the chromosome terminal of PG1 and metacentric region of PG7. This phenomenon was consistent with the fact that the translocation breakpoint is located in the distal region of PG8 and centromeric region of PG6 in peach [58]. We thus speculate that the majority of ancestral PG1 and PG7 form PM1 and PM7. The remaining mini-chromosomes fuse to form PM2. These results collectively indicate that the Prunus genome is highly conserved within the genus and may show some of the molecular genetic basis of the intercrossability and interspecific hybrid fertility among many species within this genus. However, due to the limited density and coverage of the current framework physical map, the syntenic relationships established have a low resolution. In a next step, more scaffolds will be needed to be anchored to each LG of mei, aimed at a more comprehensive analysis of genomic structure and organization in Prunus.

### Conclusions

Mei has been increasingly used as an ornamental plant in East Asia. In this study, we conducted the genome-wide characterization of SSRs in the mei genome and used SSR markers to construct a framework linkage map for mei. By analyzing the frequency and distribution of SSRs in the mei genome and comparing the pattern of SSRs among mei and other Rosaceae species and a broad range of eudicots, we have gained better insight into the evolutionary dynamics of SSRs in Rosaceae plants. The framework genetic map of mei constructed may facilitate the genetic mapping of quantitative trait loci associated with horticulturally important species-specific traits, such as cold tolerance, flower type, and flower scent. Synteny analysis has provided important clues for the reconstruction of the picture of genome speciation of Prunus species.

## Materials and Methods

### Plant Materials and DNA Extraction

A segregating F_1_ population consisting of 190 individuals (Voucher specimen accession number: BJFU1210120025-0214) derived from a cross between *P. mume* ‘Fenban’ (BJFU1210120013) and *P. mume* ‘Kouzi Yudie’ (BJFU1210120022) was used to construct a linkage map. ‘Fenban’ and ‘Kouzi Yudie’ were both advanced selections from the Qingdao Meiyuan, Qingdao, China (36°04′N, 120°20′E). Plant materials were grown in the Xiao Tangshan horticultural fields (40°02′N, 115°50′E) affiliated to Beijing Forestry University, Beijing, China. Total DNA was extracted from fresh young leaves with the plant genomic DNA extraction Kit (TIANGEN, Beijing, China) following the manufacturer’s instructions.

### Data Mining for Genome Sequences Containing SSRs

Genome sequences of Arabidopsis, cocoa, pigeon pea, Chinese cabbage and potato were downloaded from TAIR database (version 10) (http://www.arabidopsis.org/), CocoaGen database (version 2) (http://cocoagendb.cirad.fr/), IIPG database (http://www.icrisat.org/gt-bt/iipg/Genome_Manuscript.html), Brassica database (version 1.2) (http://brassicadb.org/brad/) and PGSC database (version 3 2.1.10) (http://potatogenome.net/), respectively. Strawberry (version 1.1) and apple (version 1.0) genome sequences were downloaded from GDR (http://www.rosaceae.org/). The whole-genome sequences of mei have been recently obtained by Solexa sequencing in the authors’ lab and were downloadable from the Mei genome database (http://prunusmumegenome.bjfu.edu.cn./). We have uploaded the genome assembly to the NCBI Bio-project under accession PRJNA171605 and have deposited the raw data at NCBI Sequence Read Archive (SRA) under accession SRA056478 (de novo).

Computer program MISA (MIcroSAtellites identification tool, http://pgrc.ipk-gatersleben.de/misa) was used to scan for perfect SSRs (single motif in an uninterrupted array) against each of the plant genome. Minimum repeat lengths for SSR findings were set to 10 bp for mononucleotides, 12 bp for di- to tetranucleotides, 15 bp for pentanucleotides, 18 bp for hexanucleotides, 21 bp for heptanucleotides and 24 bp for octanucleotides.

### Annotation of SSR-containing CDS in the Mei Genome

Each CDS containing SSR motifs was aligned to TAIR dataset (version 10) (http://www.arabodopsis.org) using BLASTx (E <10^−15^) and the orthologous sequences of mei were assigned functional annotations based on the available GO tool at TAIR (http://www.arabodopsis.org/tools/bulk/go/) using Arabidopsis orthologs as input (AGI codes). The annotated sequences were mapped to high level categories (plant GO Slim) using GO Slim Viewer [59] according to the three principal GO categories (molecular function, biological process, and cellular component) by AGI codes.

### SSR Primers Design

The assembly sequences for mei genome containing SSRs were scanned by Primer 3 (version 1.1.4) to design oligonucleotide primers flanking the repeats [60]. The optimized input parameters for the Primer 3 software in this study were: amplicon size (minimum, optimum, maximum): 100-250-400 bp; primer size (minimum, optimum, maximum): 18-22-27 bp; primer Tm (minimum, optimum, maximum): 45-55-65°C; primer GC content (minimum, optimum, maximum): 30–40–60%; CG clamp: 0; maximum end stability: 250; maximum Tm difference: 2; maximum self-complementarity: 6; maximum 3′ self-complementarity: 3; maximum Ns accepted: 0; maximum poly-X: 5.

### SSR Primer Screening and PCR Amplification

Six hundred and seventy pairs of SSR primers (Table S8) were screened for polymorphisms between the two parental lines and among five randomly chosen segregating progeny using PAG electrophoresis. The PCR amplification reactions were conducted in a total volume of 25 µl containing 100 ng of genomic DNA, 2.5 µl of 10×buffer [20 mM Tris-HCl (pH 8.4), 20 mM KCl, 10 mM (NH_4_)_2_SO_4_, and 1.5 mM MgCl_2_], 1.8 µl of 2.5 mM dNTP, 1.8 µl of 10 µM each of forward and reverse primers, 1.5 U of Taq DNA polymerase (Promega, Madison, WI, USA), and ddH_2_O to the total volume. The PCR conditions were as follows: 4 min at 95°C, followed by 35 cycles of 30 s at 95°C, 40 s at the optimal annealing temperature for each primer pair (Table S8), 1 min at 72°C, and an 8 min final extension at 72°C. Each PCR product was run on 1% agarose gel at 100 V and then was separated by 6% denaturing PAG electrophoresis with 1×TBE buffer at 80 W for 110 min. The gels were visualized using silver staining in accordance with the detailed protocol [61].

Polymorphic SSR primers were labeled with fluorescent dyes and amplified in the parental lines and 190 segregating progeny. SSR genotyping was carried out using a three-primer strategy, including a forward primer labeled with FAM, HEX or TAMRA (Beijing Microread Genetics Co., Ltd, Beijing, China) and a regular reverse primer. Regardless of forward primer labeled with fluorescent dyes, 50 ng of genomic DNA, 1 µl of 10×buffer (as described above), 1 µl of 2.5 mM dNTP, and 0.8 U of Taq DNA polymerase (Promega, Madison, WI, U.S.) were applied for all three-primer PCR reactions. Depending on different fluorescent dyes tagged, the amount of primers used in three-primer PCR were 1 µl of 10 µM each of forward and reverse primers for FAM, 0.8 µl for HEX and 1.2 µl for TAMRA. Double distilled water was applied to reach a final volume of 10 µl. The three-primer PCR conditions were as follows: 4 min at 95°C followed by 30 cycles of 30 s at 95°C, 50 s at each primer’s optimized annealing temperature (Table S8), and 30 s at 72°C and a final step of 6 min at 72°C. The PCR products of the three fluorescent dyes were resolved on an ABI 3730 fluorescent analyzer (Applied Biosystems, Foster City, CA, USA). ROX 400 HD served as a size standard. Data were analyzed using GeneMapper software (version 3.7) to ascertain the sizes of SSR alleles (Applied Biosystems, Foster City, CA, USA).

### Construction of a Genetic Linkage Map

Genetic linkage was analyzed using JoinMap version 4 [62] under the cross pollinator (CP) population model and regression mapping algorithm. The chi-square test (χ^2^) was carried out to test deviation of polymorphic markers from Mendelian inheritance ratios (*P = *0.05) and the region with two or more adjacent loci revealing skewed segregation (*P*<0.05) was identified as a SDR [44], [47]. Map distances were calculated according to Kosambi’s mapping function [63] and denoted in centiMorgans (cM). Markers were placed onto LGs under a likelihood odds (LOD) ratio of 5.0, and then eight LGs paralleling to the haploid chromosome number of the mei genome were determined.

### Syntenic Analysis between the Genomes of Mei and Prunus

We used the mapped markers to anchor and orient the genome assembly sequences of mei. More than one marker was present on each scaffold, allowing us to orient the scaffolds correctly and anchored them to LGs in the forward or reverse direction according to the order of corresponding markers. However, only one marker was located in each scaffold, which was considered as uncertain orientation.

Six-hundred and thirteen RosCOS markers from Prunus T×E reference map [21] were downloaded from the NCBI database and were BLASTed against the mei genome sequences anchored in the genetic map using Blat [64]. Sequences were considered orthologous when fitting the following criteria: match length ≥11 bp, Blat score ≥30, sequence identity ≥80%, alignment coverage ≥80%. Finally, a set of 192 RosCOS markers was used to analyze macro-colinearity. The Circos software package [65] was used to visualize the syntenic relationships. As the input data for Circos, bin map positions from the Prunus T×E reference map were transformed into physical positions by multiplying the sizes of the markers in cM by 100 Kbp and markers were spaced at 10 Kbp nucleotide intervals in each bin using the method described by Vladimir et al. [19]. The bin map distances of the Prunus T×E reference map were then comparable with the physical distances on the framework physical map of the mei genome. The chromosomal rearrangements between the genomes of mei and Prunus were identified using the method described by Vilanova et al. [57]. The correspondence of markers from two or more chromosomes of one species to a single chromosome of another species implied that fission, fusion, and translocation events have been found since the differentiation of the two species [57]. Any stretch with one breakpoint per translocation was assumed to contain two or more homologous sequences.

## Supporting Information

Figure S1 Relative frequency (%) of SSRs in the mei genome with respect to motif lengths. The chart is based on 188,149 SSRs identified in assembly sequences of mei genome (∼237 Mb).
(DOC)

Figure S2 GO classification of 4,089 CDS containing SSRs according to three top-level ontologies.
(DOC)

Figure S3 Polyacrylamide gel electrophoresis of SSR alleles amplified in parental line and five segregating progeny using four primer pairs. The eight samples in each pair primer (from left to right) is successively female parent, male parent, progeny 1, progeny 2, progeny 3, progeny 4 and progeny 5. M: Maker DL2000. PMSSR0009 and PMSSR0012 are polymorphic loci. PMSSR0013 is non-polymorphic locus. PMSSR0022 is no-amplified locus.
(DOC)

Figure S4 Examples of polymorphic SSR primers labeled by three fluorescent dyes resulted from mei. The blue, green and black colors respectively represent the forward primers labeled with fluorescent dyes including FAM, HEX or TAMRA. Panels show data from ‘Fenban’ (FB), ‘Kouzi Yudie’ (KZYD), and their F_1_ hybrids (HB): (A) heterozygous loci in the ‘Fenban’, two alleles; (B) heterozygous loci in the ‘Kouzi Yudie’, two alleles; (C) heterozygous loci in the parental line, two alleles; (D) heterozygous loci in parental line, four alleles; (E) heterozygous loci in parental line, three alleles.
(DOC)

Table S1 Functional annotations of 4,089 CDS containing SSR repeat motifs in mei.
(XLS)

Table S2 Distribution of perfect SSRs in genomic sequences of the other eudicots.
(XLS)

Table S3 Distribution of different mono-, di-, tri-, and tetranucleotide repeats analysed in genomes of mei and other eudicots.
(XLS)

Table S4 Distribution of different mono-, di-, and trinucleotide repeats analysed in CDS regions of mei and other Rosaceae species.
(XLS)

Table S5 Distribution of different mono-, di-, and trinucleotide repeats analysed in intergenic regions of mei and other Rosaceae species.
(XLS)

Table S6 Distribution of repeat motifs in CDS regions of mei and other Rosaceae species.
(XLS)

Table S7 Distribution of repeat motifs in intergenic regions of mei and other Rosaceae species.
(XLS)

Table S8 Details of 670 SSR primer pairs in mei genomic sequences.
(XLS)

Table S9 Distribution of SSR repeat motifs in eight chromosomes of mei.
(XLS)

Table S10 Syntenic relationships of RosCOS markers mapped to the mei pseudochromosomes.
(XLS)
