# Supplementary material for: Genome-Wide Characterization and Linkage Mapping of Simple Sequence Repeats in Mei (Prunus mume Sieb. et Zucc.)
Source: PLoS One. 2013 Mar 28;8(3):e59562. doi: 10.1371/journal.pone.0059562 (PMC3610739; doi:10.1371/journal.pone.0059562)
Supplement: Figure S1 — Relative frequency (%) of SSRs in the mei genome with respect to motif lengths. The chart is based on 188,149 SSRs identified in assembly sequences of mei genome (∼237 Mb). (DOC) [file pone.0059562.s001.doc]

**Figure S1. Relative frequency (%) of SSRs in the mei genome with respect to motif lengths.** The chart is based on 188,149 SSRs identified in assembly sequences of mei genome (~237 Mb)*.*
