# Supplementary figures and images for: Genome-Wide Characterization and Linkage Mapping of Simple Sequence Repeats in Mei (Prunus mume Sieb. et Zucc.)
Source: PLoS One. 2013 Mar 28;8(3):e59562. doi: 10.1371/journal.pone.0059562 (PMC3610739; doi:10.1371/journal.pone.0059562)

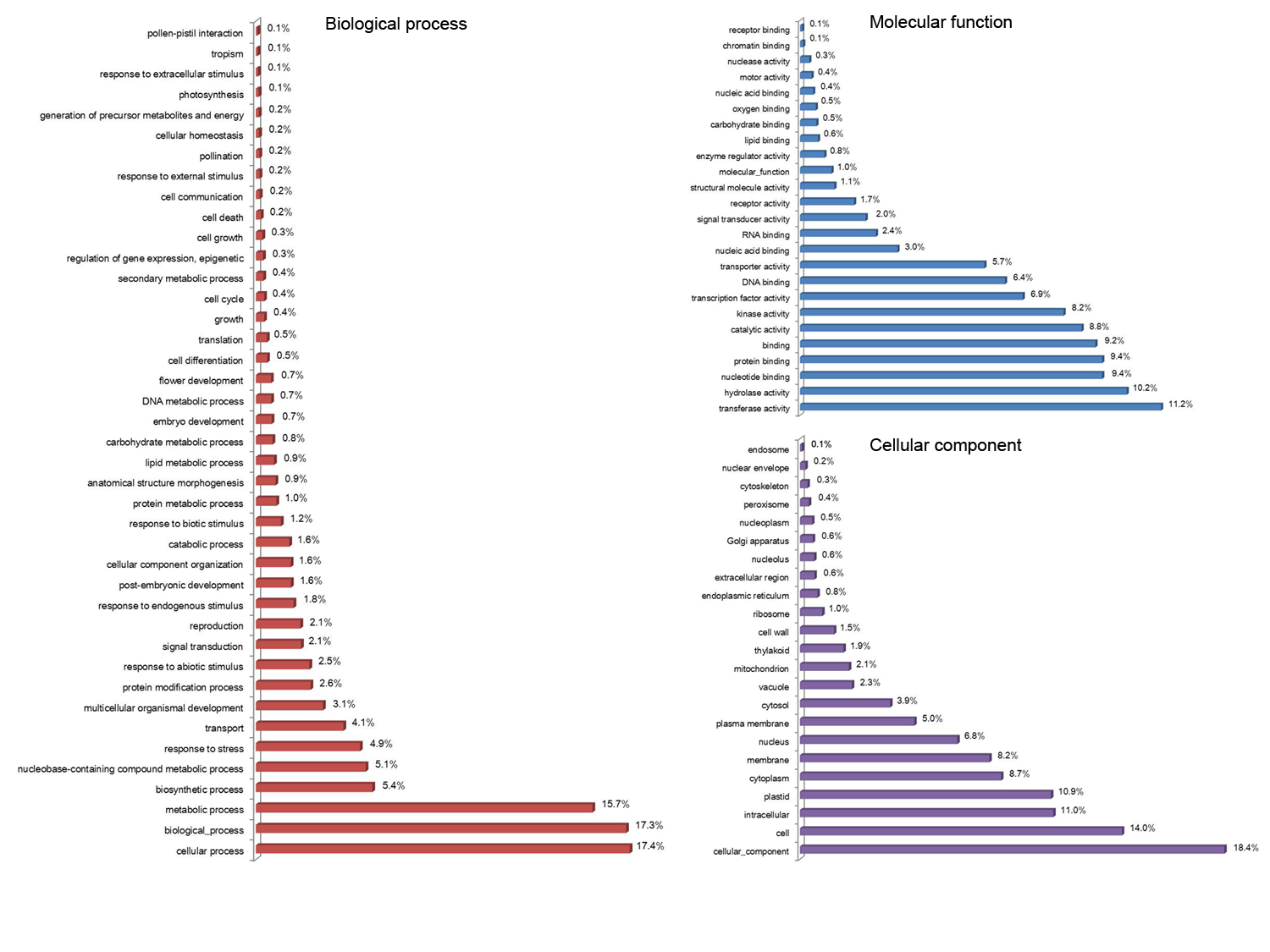


**Figure S2. GO classification of 4,089 CDS containing SSRs according to three top-level ontologies.**

Supplement: Figure S2 — GO classification of 4,089 CDS containing SSRs according to three top-level ontologies. (DOC) [file pone.0059562.s002.doc]
