# Supplementary material for: Genome-Wide Characterization and Linkage Mapping of Simple Sequence Repeats in Mei (Prunus mume Sieb. et Zucc.)
Source: PLoS One. 2013 Mar 28;8(3):e59562. doi: 10.1371/journal.pone.0059562 (PMC3610739; doi:10.1371/journal.pone.0059562)
Supplement: Figure S3 — Polyacrylamide gel electrophoresis of SSR alleles amplified in parental line and five segregating progeny using four primer pairs. The eight samples in each pair primer (from left to right) is successively female parent, male parent, progeny 1, progeny 2, progeny 3, progeny 4 and progeny 5. M: Maker DL2000. PMSSR0009 and PMSSR0012 are polymorphic loci. PMSSR0013 is non-polymorphic locus. PMSSR0022 is no-amplified locus. (DOC) [file pone.0059562.s003.doc]

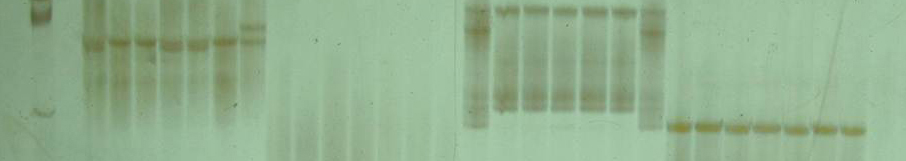


M 1 8 9 10 11 12 13 14

250bp

100bp

PMSSR0009

PMSSR0022

PMSSR0012

PMSSR0013

**Figure S3. Polyacrylamide gel electrophoresis of SSR alleles amplified in parental line and five segregating progeny using four primer pairs.** The eight samples in each pair primer (from left to right) is successively female parent, male parent, progeny 1, progeny 2, progeny 3, progeny 4 and progeny 5. M: Maker DL2000. PMSSR0009 and PMSSR0012 are polymorphic loci. PMSSR0013 is non-polymorphic locus. PMSSR0022 is no-amplified locus.
