# Supplementary material for: Genome-Wide Characterization and Linkage Mapping of Simple Sequence Repeats in Mei (Prunus mume Sieb. et Zucc.)
Source: PLoS One. 2013 Mar 28;8(3):e59562. doi: 10.1371/journal.pone.0059562 (PMC3610739; doi:10.1371/journal.pone.0059562)
Supplement: Figure S4 — Examples of polymorphic SSR primers labeled by three fluorescent dyes resulted from mei. The blue, green and black colors respectively represent the forward primers labeled with fluorescent dyes including FAM, HEX or TAMRA. Panels show data from ‘Fenban’ (FB), ‘Kouzi Yudie’ (KZYD), and their F1 hybrids (HB): (A) heterozygous loci in the ‘Fenban’, two alleles; (B) heterozygous loci in the ‘Kouzi Yudie’, two alleles; (C) heterozygous loci in the parental line, two alleles; (D) heterozygous loci in parental line, four alleles; (E) heterozygous loci in parental line, three alleles. (DOC) [file pone.0059562.s004.doc]

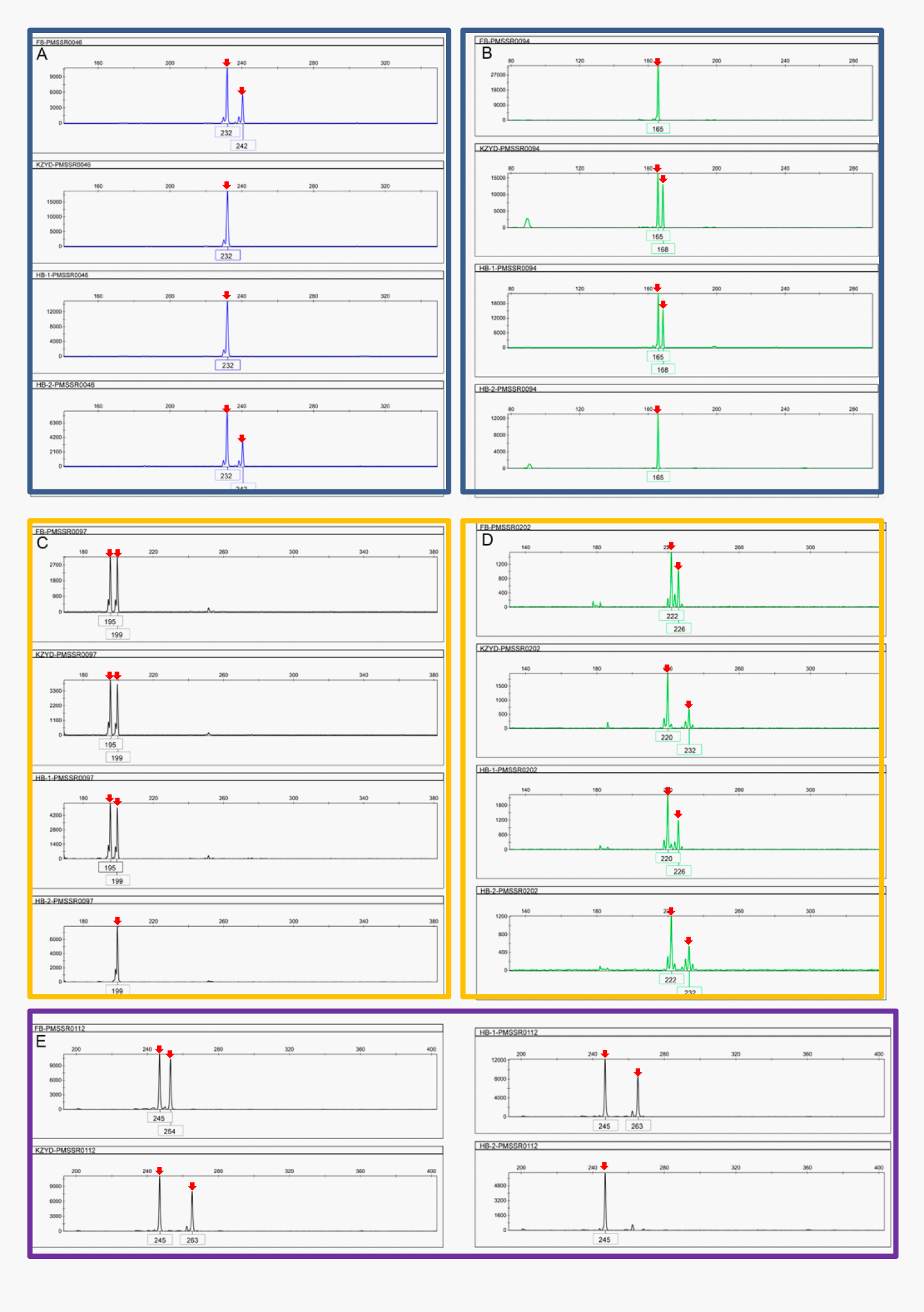


**Figure S4. Examples of polymorphic SSR primers labeled by three fluorescent dyes resulted from mei.** The blue, green and black colors respectively represent the forward primers labeled with fluorescent dyes including FAM, HEX or TAMRA. Panels show data from 'Fenban' (FB), 'Kouzi Yudie' (KZYD), and their F_1_ hybrids (HB): (A) heterozygous loci in the 'Fenban', two alleles; (B) heterozygous loci in the 'Kouzi Yudie', two alleles; (C) heterozygous loci in the parental line, two alleles; (D) heterozygous loci in parental line, four alleles; (E) heterozygous loci in parental line, three alleles.
